# Cutaneous mucormycosis in a patient with Hailey-Hailey disease (benign familial pemphigus) - first reported case

**DOI:** 10.1016/j.mmcr.2026.100819

**Published:** 2026-08-31

**Authors:** Joseph David Cooper, Dennis W. Adams, Lucy Studemeister, Allyson E. Sorensen, Christopher Hull

**Affiliations:** a Division of Infectious Diseases and AIDS Medicine, Santa Clara Valley Healthcare, San José, CA, 95128, USA; b Division of Infectious Diseases and Geographic Medicine, Stanford University, Palo Alto, CA, 94305, USA; c Department of Pathology, Santa Clara Valley Healthcare, San José, CA, 95128, USA; d Department of Dermatology, University of Utah School of Medicine, Salt Lake City, UT, 84112, USA

**Keywords:** Cutaneous mucormycosis, Hailey-Hailey disease, *Rhizopus*, Mucormycosis

## Abstract

This report describes a case of primary cutaneous mucormycosis in a patient with Hailey-Hailey Disease (benign familial pemphigus). The diagnosis of primary cutaneous mucormycosis was confirmed via microbiological culture as well as histopathology. Our patient improved with prompt surgical debridement and excision of infected, necrotic tissue with complementary antifungal therapy. To the best of our knowledge this is the first reported case of cutaneous mucormycosis in a patient with Hailey-Hailey Disease and contributes to the literature regarding pathophysiology, management and treatment for each of these rare entities.

## 1 Introduction

Mucormycosis (formerly known as zygomycosis) is a disease caused by ubiquitous, saprophytic Mucorales species distributed worldwide known to cause a constellation of clinical presentations including sino-orbital, rhinocerebral, sinopulmonary, gastrointestinal, musculoskeletal, osteoarticular, cutaneous and disseminated, multi-organ disease [[1], [2], [3]]. Cutaneous disease is the third most common presentation of disease and is classified as primary or secondary [[1], [2], [3]]. Hailey-Hailey disease (HHD) is rare autosomal dominant acantholytic disorder caused by mutations in the *ATP2C1* gene, and a form of erosive dermatoses. Cutaneous mucormycosis has rarely been reported in patients with other erosive dermatoses and this condition is likely an unrecognized risk factor for fungal disease. Herein we describe what we believe to be the first reported case of primary cutaneous mucormycosis in a patient with HHD. This report highlights the role of prompt recognition and diagnosis, timely surgical excision of infected necrotic tissue and complementary antifungal therapy. This report also highlights HHD as a previously unrecognized predisposing condition for cutaneous mucormycosis.

## 2 Case report

A 64-year-old female with a past medical history significant for essential hypertension, hyperlipidemia, morbid obesity, atrial fibrillation, heart failure with reduced ejection fraction and diastolic dysfunction, and HHD presented to a hospital due to dysuria, hematuria, back pain, diarrhea, and vomiting which progressed to altered mental status and difficulty in ambulation. HHD was managed with topical betamethasone dipropionate 0.05% cream to the affected areas up to twice daily during flares, with silver sulfadiazine 1% cream applied daily to open wounds, with nystatin powder twice daily to the area, along with Naltrexone 3mg by mouth nightly. Her last use of systemic prednisone was more than 18 months ago which was a tapering dose of 40mg over five days.

Vital signs at presentation included blood pressure of 114/78 mmHg, pulse of 86 beats per minute, temperature of 36°C, and respiratory rate of 18. Physical exam revealed a morbidly obese female, with altered mentation. She had scattered purpuric plaques in the deep fissure of the left inframammary fold. She also had purpuric plaques and superficial erosions of the intergluteal cleft, lower abdomen, infrapannus and lower extremities (Fig. 1).

**Fig. 1:**
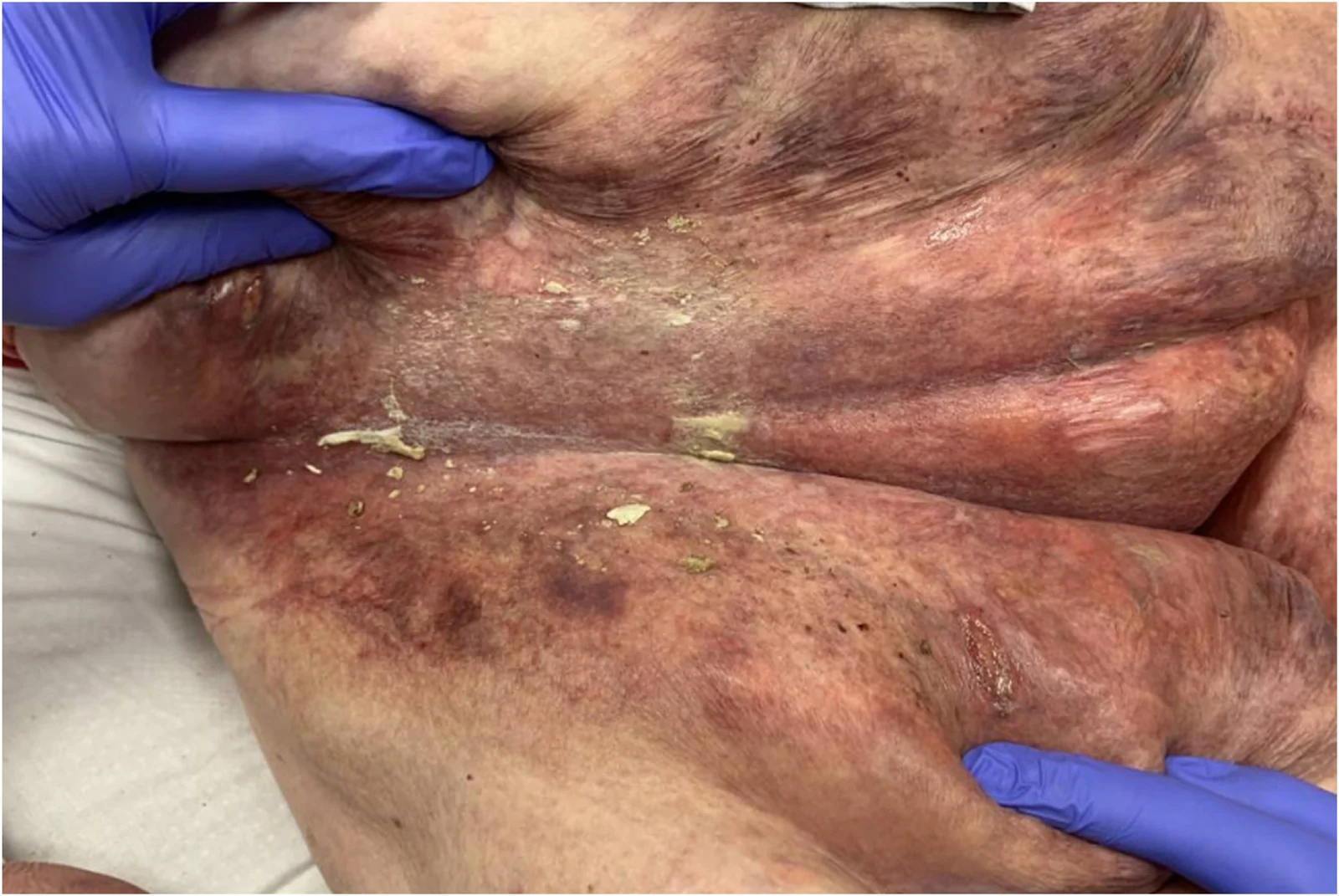
Right upper thigh, infrapannus region on hospital day 0.

Laboratory investigations revealed pyuria and hematuria, thrombocytopenia [platelet count 90 (140 - 450 x1000/mcL)], acute kidney injury [creatinine of 3.41 mg/dL (0.51 - 1.04 mg/dL)], and lactic acidosis [7.6mmol/L, (0.5 - 2.2 mmol/L)]. Two sets of peripheral blood cultures grew *Proteus mirabilis* in two out of four bottles, with the same organism isolated in urine cultures. Computed tomography (CT) of the chest, abdomen and pelvis without contrast revealed moderate left hydronephrosis with a 6mm obstructing stone at the left proximal ureter and a non-obstructing 5mm stone at the left inferior renal pole. She was admitted to the intensive care unit (ICU) and received vasopressors, empiric antimicrobials vancomycin and meropenem, and hydrocortisone 50mg intravenously (IV) every 6 hours for four days.

On hospital day 1 she underwent cystoscopy with placement of a left ureteral double J stent (JJ) per Urology. Intra-operative findings revealed an inflamed bladder wall with debris and copious efflux of purulent fluid following stent placement. She completed a fourteen-day course of appropriate antimicrobials and was transferred to a general medical floor. Dermatology was consulted on hospital day 6 due to worsening ecchymosis and evolving eschars of her wounds. On hospital day 11 a bacterial wound Gram stain and culture was collected from the right infrapannus abdominal region which revealed few polymorphonuclear leukocytes (PMN's) without organisms. Final bacterial culture results were negative. Herpes simplex virus (HSV)-1 nucleic acid was detected from the same location and valacyclovir 1 g by mouth twice daily was administered for a total of three weeks.

Due to hypotension, tachycardia and worsening appearance of the scattered erosions and eschars on the groin and pannus, a surgical consultation was requested (Fig. 2). A repeat CT scan of the abdomen and pelvis without contrast did not reveal a deep tissue abscess or fluid collection. On hospital day 19, plastic surgery performed a bedside excision and biopsy at the right sided dependent pannus and upper thigh. Eschar and dead tissue were removed via electrocautery measuring 7x4x2 cm total. Tissue was evaluated for histopathology and microbiological analysis.

**Fig. 2:**
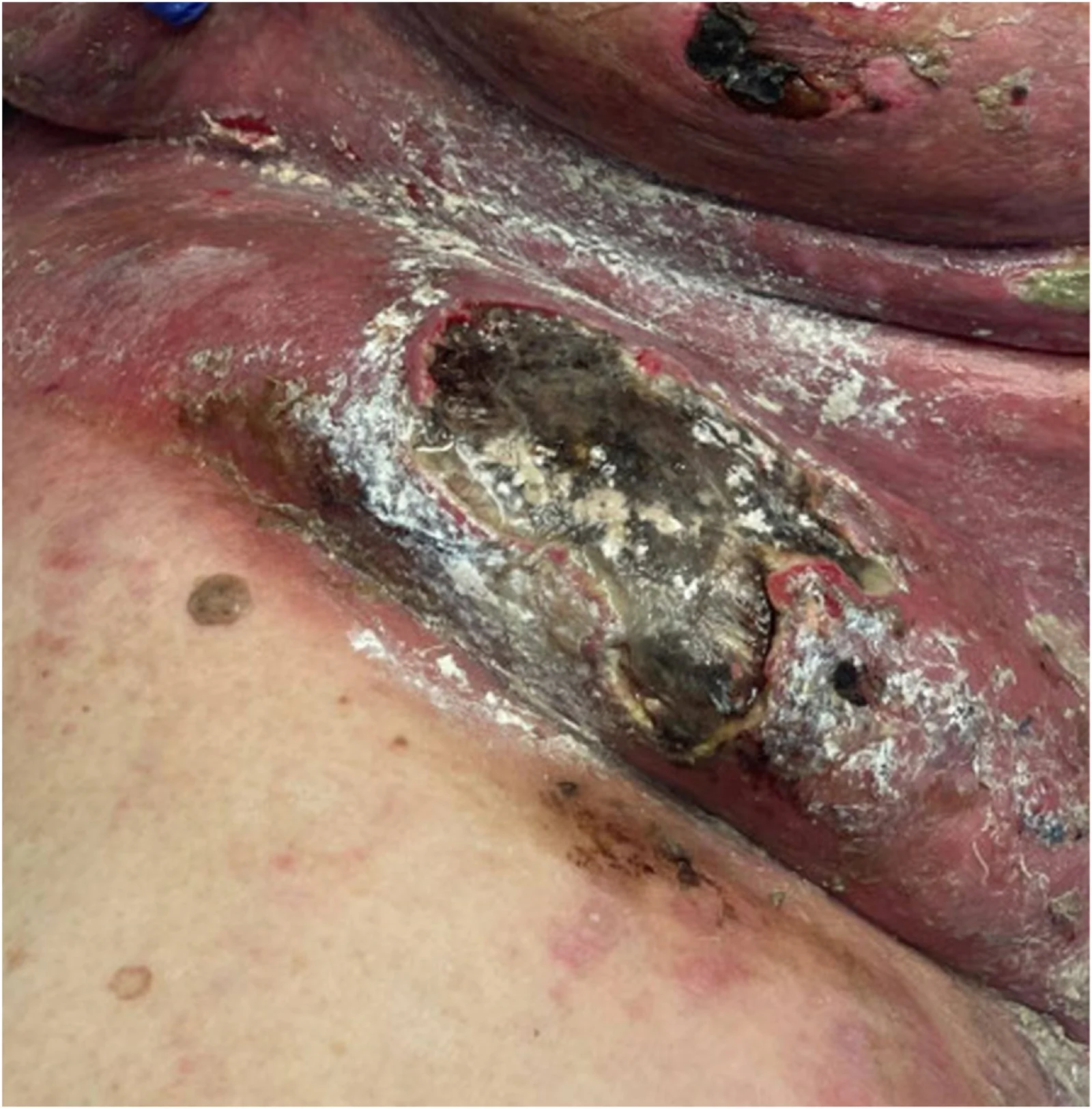
Right upper thigh with large ulceration, eschar and surrounding erythema and induration on hospital day 19.

Gram stain revealed no PMN's and showed moderate hyphal elements. Identification of a filamentous fungi, *Rhizopus* species was made after the specimen was sent to a reference laboratory. Antifungal susceptibilities were as follows: Amphotericin MIC (μg/mL): 1, Isavuconazole: 2, Itraconazole: 4, Posaconazole: 0.5, Fluconazole: resistant (no MIC listed), Voriconazole (no MIC listed). Gross pathological examination of tissue revealed gray to yellow-brown necrotic fatty tissue covered in pus. Histopathology revealed an acantholytic dermatosis with necrosis, suppuration, and angioinvasive fungi, consistent with invasive mucormycosis. Grocott-Gomori methenamine silver (GMS) and periodic acid-Schiff (PAS) stains visualized large, broad, ribbon-like aseptate hyphae with right-angle branching (Fig. 3).

**Fig. 3:**
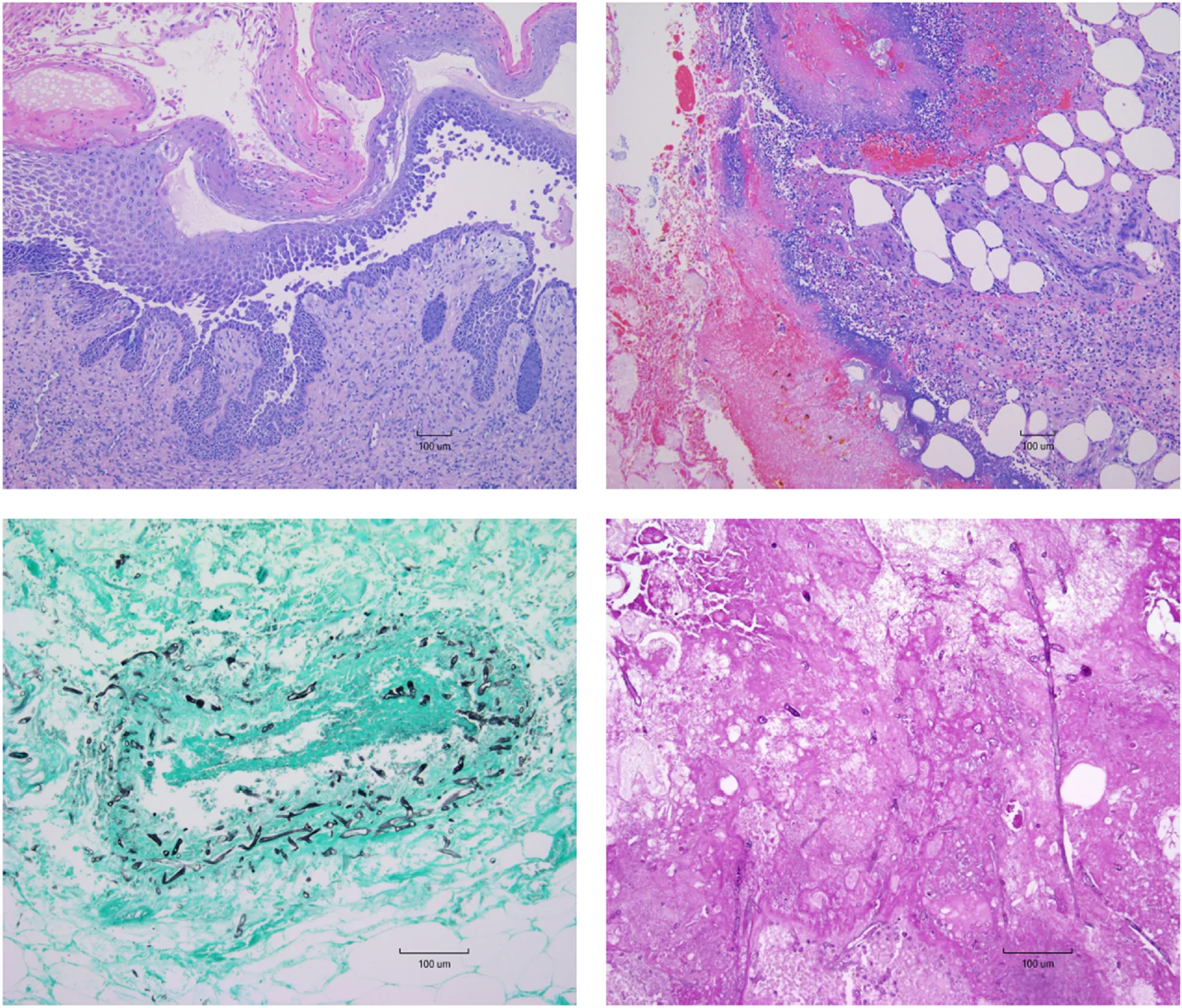
Histopathology of acantholytic dermatosis consistent with Hailey-Hailey disease (top left, H&E, 100x) and necrosis with inflammatory exudate (top right, H&E, 100x). GMS stain (bottom left, 200x) and PAS stain (bottom right, 200x) illustrating large, broad, ribbon-like aseptate hyphae with right-angle branching.

Liposomal amphotericin B at 5mg/kg IV daily along with posaconazole delayed-release tablet 300mg by mouth twice daily for two doses followed by 300mg by mouth once daily was initiated. On hospital day 23, she was taken to the operative suite by plastic surgery for debridement of the abdomen, bilateral inguinal folds and bilateral breast soft tissue and fat. Intra-operative findings were significant for necrotic tissue and eschar on abdomen, thighs, groins and chest which were debrided and removed. Right thigh wound tissue was sent for microbiological analysis and histopathology, including tissue for margins. Gram stain revealed no PMN's and showed moderate hyphal elements. Identification of *Rhizopus* species was made; antifungal susceptibilities were not obtained on this specimen. Histopathology revealed acantholytic dermatosis with dermal chronic inflammation and fat necrosis. No fungal organisms were observed on PAS stain.

Dual antifungals were continued, and liposomal amphotericin B was discontinued after a total of two weeks due to acute kidney injury. Posaconazole was increased to 400mg by mouth daily, due to an initial subtherapeutic level, and a level of 1.9 μg/mL (reference range≥0.8 μg/mL) was achieved. Local wound care and a wound vacuum were utilized for treatment. No further debridement was indicated due to source control (Fig. 4), and she was discharged on hospital day 59 to a long-term acute care hospital. Outpatient follow up more than 2 months after surgical debridement revealed a healing right thigh wound and total duration of antifungal therapy of 3 months from surgical debridement was recommended (Fig. 5).

**Fig. 4:**
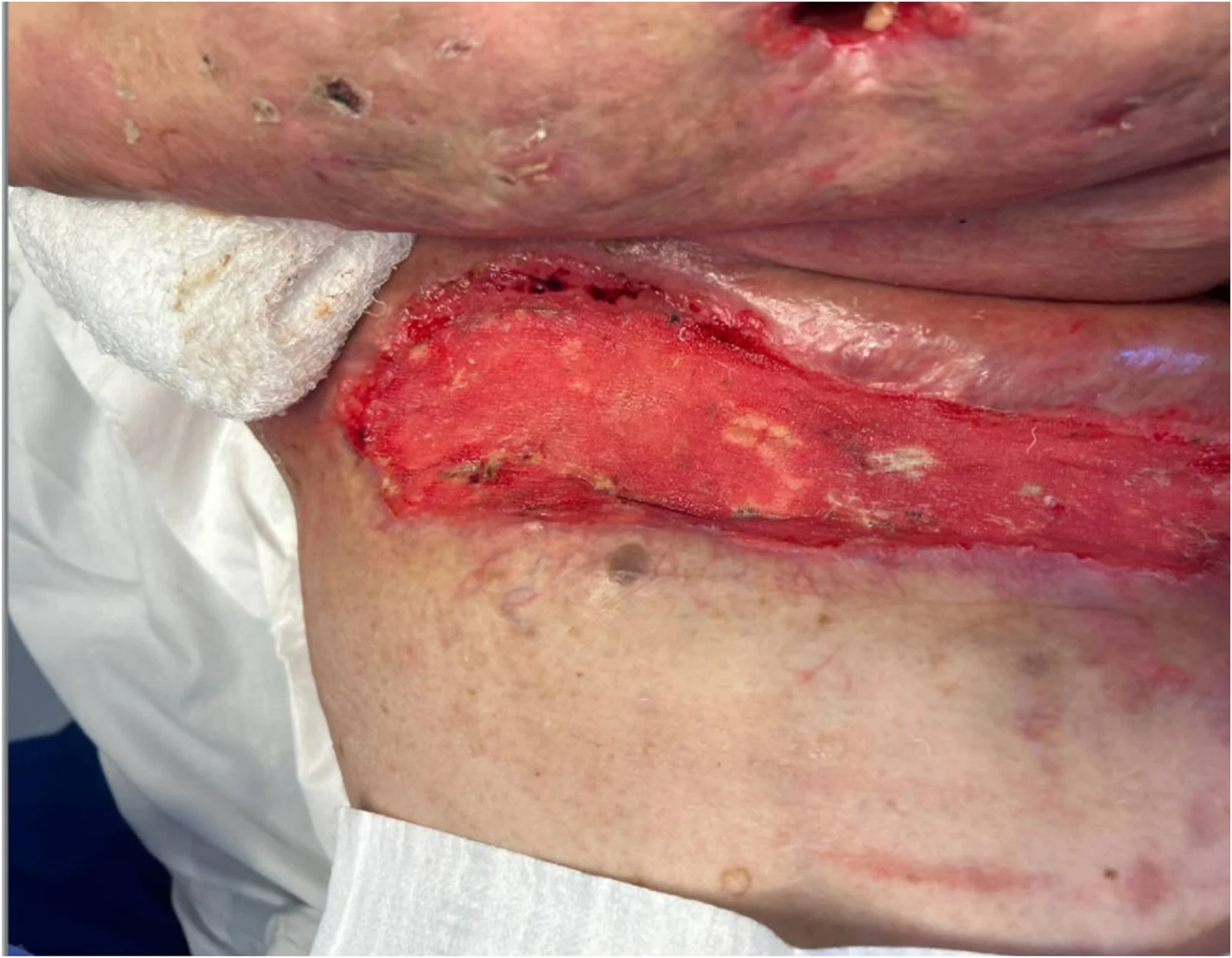
Right upper thigh after surgical debridement on hospital day 59 prior to discharge.

**Fig. 5:**
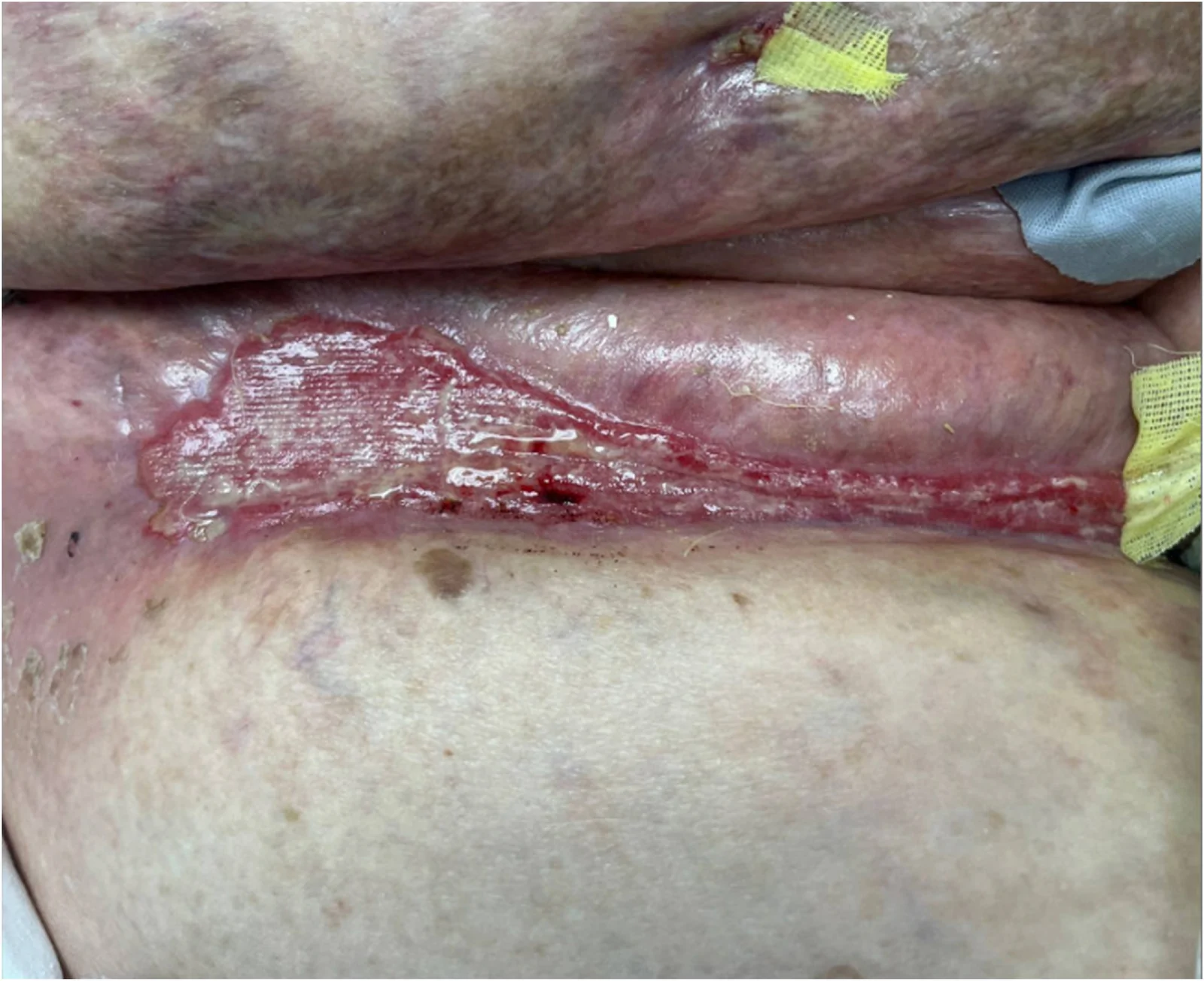
Right upper thigh more than 2 months after initial surgical procedure.

## 3 Discussion

Mucormycosis is a broad term, first described in 1885 as mycosis mucorina, which are caused by members of the order Mucorales, within the subphylum Mucormycotina [[1], [2], [3]]. Mucorales species are saprophytic molds that are found ubiquitously in soil and decaying organic matter in the environment, worldwide [2,3]. Human disease usually occurs after inhalation of sporangiospores (mycelium growing in nature) 3 to 11 μm in diameter via the nasal and respiratory tract and less commonly via cutaneous acquisition due to disruptions in the skin barrier [4]. Cutaneous is the third most common presentation of disease; however, the incidence is increasing [5,6]. Cutaneous disease is typically classified as primary or secondary.

Primary cutaneous disease results after direct inoculation of the skin from trauma, injury or major burns leading to “immune paralysis,” mechanistically described as functional neutropenia due to mediators released from damaged tissue causing local neutrophil and immune dysfunction [2,7]. Nosocomial transmission due to contaminated bandages, or elastic/adhesive tape at catheter or venipuncture sites has been reported [2,7]. Secondary cutaneous disease occurs due to dissemination to the skin, typically in immunocompromised patients [6,7]. Immunocompromising conditions predisposing to disease include uncontrolled diabetes mellitus, receipt of prolonged systemic corticosteroids, solid organ or bone marrow transplantation, hematological malignancies, metabolic acidosis and neutropenia [8,9].

Typical symptoms of cutaneous disease include local pain at the site of injury usually within 1 to 8 days; however, one study showed delayed presentation for up to seven years after traumatic event [10]. The most common affected areas of the skin are the arms and legs; however, the disease has been observed in the scalp, face, thorax, back, abdomen, perineum, breast, neck and gluteal areas as well [11]. Morphology varies; lesions are typically limited and indurated and may range from erythematous to purple [7,11]. Lesions may evolve into necrosis, and an erythematous halo may develop into an eschar [7,11]. Atypical cutaneous manifestations include targetoid lesions, tender nodules, ulcers, purpuric lesions and scaly plaques [11]. Patients with underlying erosive dermatoses may be at risk for cutaneous mucormycosis as well [12]. Due to its angioinvasive nature, cutaneous vasculature infarction occurs causing a brown to black discoloration/eschar with a firm, woody consistency, at times with draining exudates [2,4,13]. Cutaneous disease progression may result in necrotizing fasciitis, sinus tracts, muscle and tendon involvement, even osteomyelitis [4,7].

Cutaneous differential diagnoses include autoimmune diseases, drug reactions, infiltrative diseases, pyoderma gangrenosum and other invasive fungal infections such as aspergillosis, fusariosis, ecthyma gangrenosum caused by *Pseudomonas aeruginosa* or other Gram-negative bacilli [2,7]. Early diagnosis and initiation of antifungal therapy is paramount and significantly reduces morbidity and mortality [2,3]. Early identification may be achieved by direct potassium hydroxide (KOH) microscopic examination which illustrates numerous coenocytic, ribbon-like, nonseptated hyphae that are broad (typically 7 to 15 μm in diameter) with nondichotomous branching [2,7]. Mucorales species typically grow rapidly as a floccose colony on Sabouraud's glucose agar between 25 and 37°C and *Rhizopus* species is the most isolated Mucorales species [14]. Other laboratory tools are increasingly being used for the diagnosis including nucleic acid amplification testing (with quantitative PCR assays to target the 18S ribosomal RNA) on a variety of clinical specimens as well as metagenomic analysis of cell-free DNA [2].

Biopsy and histopathological analysis are critical in the diagnosis and management of cutaneous mucormycosis. Detection of characteristic broad, ribbon-like, nonseptated or sparsely septate fungal hyphae with right-angle branching in tissue samples stained with PAS and GMS stains establishes the diagnosis [2]. Histopathology typically reveals an inflammatory reaction with edema, necrosis, and an abundance of polymorphonuclear and plasma cells with occasional eosinophils [5]. Thrombosis and tissue infarction are commonly seen due to fungal angioinvasion [5]. Examination of routine hematoxylin and eosin (H&E) stains of formalin fixated, paraffin embedded tissue sections can potentially miss the diagnosis and immunohistochemical techniques or fluorescence in situ hybridization may be utilized in some laboratories to establish a microbiological diagnosis [2].

Treatment success largely depends on prompt diagnosis, initiation of antifungal therapy, and surgical debridement and removal of all infected and necrotic tissue [2,7]. Although randomized, controlled trials including patients with mucormycosis are overall lacking, amphotericin B is the cornerstone antifungal therapy [2,3,9]. Triazole antifungals are considered for salvage therapy, and isavuconazole is the only licensed triazole for primary treatment [2]. Posaconazole has shown efficacy as salvage therapy as well [2]. Recent innovative approaches for treatment of cutaneous disease include Mohs surgery and hyperbaric oxygen as adjunctive therapy [15,16].

To the best of our knowledge, this is the first reported case of cutaneous mucormycosis in a patient with HHD. Lu et al. detail two cases of primary cutaneous mucormycosis as a complication of erosive dermatitis in patients with psoriasis, erosive erythroderma and toxic epidermal necrolysis (TEN)/Lyell syndrome [12]. Jin et al. also describe a case of TEN complicated by mucormycosis [17]. Our patients’ risk factor for acquisition of disease may be secondary to her underlying HHD which is a form of erosive dermatitis, resulting in primary cutaneous disease due to a disrupted skin barrier. It is unclear whether mucormycosis was acquired prior to admission or nosocomially.

HHD is a rare autosomal dominant acantholytic disorder caused by mutations in the *ATP2C1* gene, which is essential for keratinocyte adhesion [[18], [19], [20]]. The resulting impairment of desmosomal integrity produces chronic, recurrent vesicles, erosions and macerated plaques that characteristically involve intertriginous sites including the axillae, inframammary folds, groin, and intergluteal cleft, the same regions affected in our patient. This persistent disruption of the cutaneous barrier, compounded by the warm, moist, and frequently macerated microenvironment of these flexural areas, predisposes the skin to secondary bacterial, candidal, and viral superinfection (including HSV, as observed in our patient) and plausibly provides a portal of entry for environmental Mucorales [[18], [19], [20]]. Taken together with prior reports of primary cutaneous mucormycosis arising in other erosive dermatoses, these features support HHD as a previously unrecognized predisposing condition for cutaneous mucormycosis [12,17].

Another risk factor in her acquisition of disease was receipt of IV hydrocortisone for four days, a well described risk factor for developing mucormycosis. There is a paucity of data regarding the risk of cutaneous mucormycosis with the chronic use of topical corticosteroids, but to the best of our knowledge this is not well described in the literature and less likely contributory. Our patient was successfully treated with prompt surgical debridement and excision of infected necrotic tissue with complementary antifungal therapy. Optimal duration of therapy for cutaneous mucormycosis is also not well described in the literature and warrants further investigation.

## CRediT authorship contribution statement

**Joseph David Cooper:** Conceptualization, Data curation, Investigation, Supervision, Writing – original draft, Writing – review & editing. **Dennis W. Adams:** Data curation, Writing – review & editing. **Lucy Studemeister:** Writing – review & editing. **Allyson E. Sorensen:** Data curation, Writing – review & editing. **Christopher Hull:** Data curation, Supervision, Writing – original draft, Writing – review & editing.

## Consent for publication

Written informed consent was obtained from the patient for the publication of this case report.

## Funding

This research did not receive any specific grant from funding agencies in the public, commercial or not-for-profit sectors.

## Declaration of competing interest

The authors declare no competing interests.
